# Self-perceived uselessness is associated with lower likelihood of successful aging among older adults in China

**DOI:** 10.1186/s12877-016-0348-5

**Published:** 2016-10-06

**Authors:** Danan Gu, Bethany L. Brown, Li Qiu

**Affiliations:** 1 United Nations Population Division, Two UN Plaza, DC2-1910 New York, USA; 2 Health and Human Rights Division, Human Rights Watch, New York, USA; 3 Independent Researcher, New York, USA

**Keywords:** Self-perceived uselessness, Self-ageism, Negative perceptions, China, Usefulness, Psychological resilience, Successful aging, Older adults, Oldest-old

## Abstract

**Background:**

Plenty of evidence has shown that self-perceived uselessness among older adults is negatively associated with successful aging in terms of good health in Western societies. It is unclear whether these findings are valid in China where living into older age is more selective due to high mortality at younger ages.

**Methods:**

Using five waves (2000, 2002, 2005, 2008/2009 and 2011/2012) of a large nationally representative survey in China with 29,954 observations from 19,070 older adults aged 65 and older, this study aimed to investigate the association between self-perceived uselessness and successful aging. Self-perceived uselessness was measured by a single item “with age, do you feel more useless?” with six answers: always, often, sometimes, seldom, never, and unable to answer. Successful aging was measured by independence in activities of daily living (ADL), independence in instrumental activities of daily living (IADL), unimpaired cognition, good life satisfaction, and good self-rated health. Logistic regression models were applied to each successful aging indicator after controlling for a rich set of covariates that included demographics, socioeconomic status, family/social support, and health practices. The models also adjusted for intraperson correlations across waves.

**Results:**

We found that self-perceived uselessness was negatively associated with successful aging among older adults aged 65 or older. Specifically, compared to never having self-perceived uselessness, always having such a perception was associated with 16–42 % lower odds of being ADL independent, IADL independent, cognitively unimpaired, and having good life satisfaction and good self-rated health. Often or sometimes having such a perception also reduced odds of aging successfully, although such reductions were less pronounced. The associations were similar among the oldest-old aged 80 or older with one exception for the case of IADL independence.

**Conclusions:**

Self-perceived uselessness is negatively associated with successful aging among Chinese older adults as well as among the oldest-old. Our findings could be informative for China in the development of public health programs that aim to improve self-perceptions about aging and promote successful aging.

## Background

Self-perceived uselessness normally means an individual’s own negative assessment or perception about his or her usefulness or importance to family, friends, community, and/or the larger society and his or her general understanding of the aging process [1–4]. Self-perceived uselessness is considered a core component of self-perception of ageing or self-ageism that shapes one’s thoughts and feelings, and influences one’s behavioral patterns in older age [1–10], which could in turn adversely impact one’s psychological and physiological well-being [1, 2, 11]. In older age, a shrinking social network size and reduction in social contacts because of health deterioration would limit performance of social and family roles to which older adults may feel useful [1, 12]. Such a retreat from or reduction in frequency in social or family activities may cause self-perceived uselessness.

Plenty of evidence has consistently demonstrated that self-perceived uselessness or self-perceptions of negative aging are associated with higher risk of death [2, 3, 10, 13–16], higher rates of functional impairment and disability [1–3, 9, 17], chronic conditions [18, 19], lower rates of recovery from illness [20], poorer functions of cognition and mental health [21–23], and lower rates of self-rated good health and life satisfaction [24–27]. By contrast, many studies also reported that positive self-perceptions of aging (i.e., the absence of self-perceived uselessness) are linked with better overall survival, functional status, and life satisfaction [3, 13, 14, 28–31]. Studies further suggest that a high level of self-reported uselessness is associated with lower levels of social engagement, physical activity, self-efficacy, self-esteem, and higher levels of depression [1–4]. Feelings of uselessness may also negatively influence self-care and engagement in health promotion behaviors [1, 2, 13].

In other branches of aging studies, successful aging has gained a new momentum in the last two decades, expanding its original scope from a mainly biomedical model to a biopsychosocial model. The former defines successful aging as a state of avoidance of physical and physiological decline, absence of disability, and continued social participation/engagement [32], while the latter additionally includes psychological characteristics, life satisfaction and wellbeing, capacity for personal development, mastery and growth, positive adaptation, social networks and support, integration and participation, and a self-rating of successful aging [33–38]. There has been a developing consensus in new research that successful aging is multidimensional [33, 37–45]. This extension of the definition of successful aging has enriched our understanding of the human aging process from new perspectives, which could help to identify some possible key mechanisms of successful aging that are not yet well understood.

However, one major challenge in the studies of successful aging is the lack of a consensus about an operational definition of successful aging [33, 34, 40]. With a few exceptions that either applied the latent construct [46] or respondents’ self-ratings of successful aging [38, 43, 45, 47, 48], a common practice of most studies is to use several different health outcomes (e.g., absence of disability, cognitive impairment, psychological well-being) and self-rated life satisfaction as proxies to measure successful aging due to wide influence of the biomedical model and unavailability of data on subjective ratings of successful aging [34, 35, 40, 42, 49, 50].

One limitation in the existing literature of self-perceived uselessness is that the research has focused primarily on Western cultures and high-income countries [51, 52]. It is unclear whether the findings found in developed countries are valid in developing countries, such as in China, where older adults’ survival is more selective due to high mortality at younger ages, and the research on associations between self-perceived uselessness and health or mortality is very rare. Additionally, different cultures may also have formed different social norms about aging and have different pressures on older people.

Another limitation of the existing literature in self-perceived uselessness is that large-scale studies are still rare. Most studies in this area have had relatively small sample sizes, either from local or non-population-based studies [14, 28, 31]. This may limit the generalizability of the findings of such studies. A further limitation is that with few exceptions [1, 2, 53], most studies have not tested whether the association between self-perceived uselessness and health is still valid among the oldest-old population aged 80 or older who generally experience higher rates of frailty compared to younger older adults aged 65 to 79. In addition, with few exceptions [51], most studies have not investigated the associations between self-perceived uselessness and outcomes of successful aging. In light of the existing literature regarding the negative effect of self-uselessness on various health outcomes, this study aims to examine the association between self-perceived uselessness and successful aging among older adults in mainland China (hereafter China), a non-Western country, using a large nationally-representative sample. We hypothesize that older adults who had self-perceived uselessness in a wave were less likely to age successfully in subsequent waves in terms of ADL independence, IADL independence, cognitive without impairment, self-rated good health, and self-rated good life satisfaction. Below we provide a brief background about self-perceived uselessness among older adults in China.

### Self-perceived uselessness among Chinese older adults

The population of older adults aged 65 years or older in China accounted for 10 % of the total population in 2015. The life expectancy at age 65 was 17.6 years for women and 15.7 years for men in 2015 [54]. About 90 % of remaining life expectancy would be in active status for both men and women [55]. China has the largest older adult population in the world and it will continue to keep this ranking until possibly as late as 2080 [54]. Most older adults live in the community with children/grandchildren rather than in institutions. Less than 2 % of older adults live in institutions, mainly because of shortage of caregiving resources [56]. The proportion of older adults living alone or with a spouse only is increasing. However, currently the majority of older adults still coreside with children and/or grandchildren [57]. The retirement age is 60 years for men and 55 years for white collar women and 50 years for blue collar women. But the government aims to gradually postpone the retirement age to 65 years by 2045 for both men and women [58]. After retirement, most will provide care to grandchildren and less than 15 % will reenter back to the labor market [59]. Family is the main financial resource for rural older adults, and retirement wage is the main financial resource for urban older adults [59]. The proportion of older adults living in urban areas accounted for about 50 % of the entire older population in 2015 [59].

China has a strong filial piety tradition rooted in Confucian culture. Maltreatment of older persons is prohibited in China’s constitution. In such a context, ageism (societal negative attitude toward older adults) should not be prevalent in China. However, the filial piety tradition is fading and the decision-making power of older adults is sometimes threatened due to societal modernization and individualization of younger generations [60–62]. There is some evidence showing financial mistreatment and neglect in caregiving [63, 64].

Research on explicit measures of self-perceived uselessness in China is almost nonexistent because of the unavailability of data, although it is very frequently addressed or discussed implicitly in narrative articles. The CLHLS is the only nationwide survey that collects data on self-perceived uselessness. According to CLHLS data, about 20 % of older adults aged 65 years or older always or often feel useless; and this proportion slightly increases to 25 % among the oldest-old aged 80 years or older. Nevertheless, several studies have investigated self-perceptions of feelings of family burden, getting older, and falling behind on social progress and their associations with depression and loneliness based on some local samples [63, 64]. According to a nationwide survey on older adults, the proportion of the older population reporting feelings of being a family burden, getting older, and falling behind social progress were 50–70 % in 2000 [65], and the proportions were similar in 2010 [66]. These self-perceived feelings are components of self-perceived negative attitudes toward aging, or self-ageism [60], which is closely related to feeling of uselessness.

## Methods

### Study sample

We used data from the Chinese Longitudinal Healthy Longevity Survey (CLHLS). The CLHLS is an ongoing national survey of Chinese older adults aged 65 or older. It started in 1998 and follow-ups were implemented in 2000, 2002, 2005, 2008/2009, 2011/2012, and 2014. The first two did not include the individuals aged 65-79 years old. One major purpose of each wave of the CLHLS is to interview all centenarians in a randomly selected half of the counties/cities in 22 Han-ethnicity-dominated provinces in mainland China. The CLHLS reports that the exclusion of the non-Han minority-dominated 9 provinces is due to inaccuracy of age-reporting at old ages [67]. The 22 provinces covered about 82 % of the total population in China in 2010.

The CLHLS aims to interview all centenarians in the sampled cities/counties. Various sources whenever available were used to validate accuracy of age of centenarians, including the birth certificate, genealogical documents, household booklets, and the ages of children and siblings [67]. For each sampled centenarian, one nearby octogenarian and one nearby nonagenarian with pre-designated age and sex based on the code of the sampled centenarian were randomly interviewed (living in either a community or an institution regardless of their health conditions); and for every three sampled centenarians, four participants aged 65–79 (living in either a community or an institution regardless of their health conditions) were randomly chosen in the same cities/counties where centenarians were living at the time of survey. Each respondent provided a written informed consent to indicate his/her willingness to participate in the CLHLS. The informed consent was signed by the next-of-kin in the case when the respondent was not able to write. All information was obtained through in-home interviews about two hours in length. The response rate for each wave was around 98 % [38]. Detailed sampling procedures can be found in elsewhere [67, 68].

**Fig. 1:**
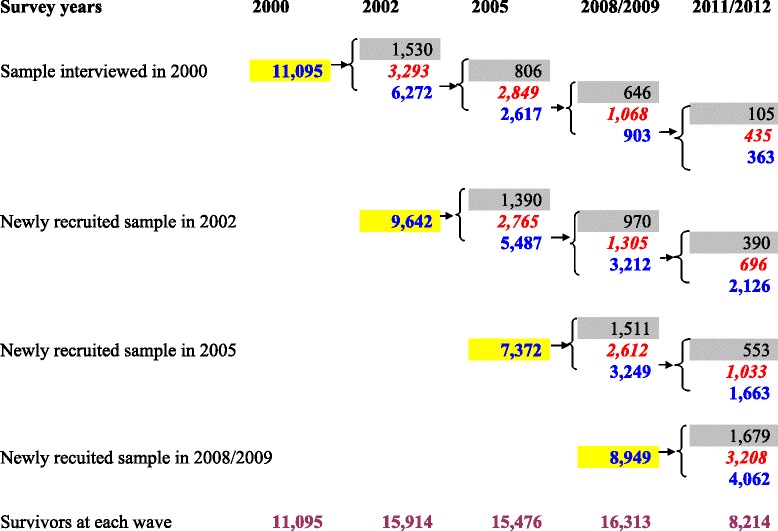
Structure of sample by survey year, initial interview year, and survival status at a follow-up wave. (1) Figures shaded in *grey* were those lost to follow-up. They are excluded from the present study. Figures in italic *red* font were deceased persons who died before a follow-up, and were also excluded. Figures shaded in *yellow* were newly recruited samples at a survey. Survivors in a follow-up wave are represented by blue font. (2) The total valid number of individuals who had at least one follow-up interview was 19,070 (=6272 + 5487 + 3249 + 4062)

Because the wording of the responses to self-perceived uselessness in the 1998 wave was not the same as the following waves and because the 2014 wave was not available when this study was started, we relied on the 2000, 2002, 2005, 2008/2009, and 2011/2012 waves. A great deal of evidence shows that the data quality of the CLHLS is quite good [67]. The summarized distribution of the interviewed respondents in each wave and their follow-up status is presented in Fig. 1. The study sample in this research consisted of 19,070 respondents with at least one follow-up who contributed 29,954 observations in total from 2000 to 2011/2012.

### Measurements

#### Self-perceived uselessness

Self-perceived uselessness was measured by a single question, “with age, do you feel more useless?” The question has six response options: always, often, sometimes, seldom, almost never or never, and unable to answer. In order to keep as much as information in its original form for the analysis, we did not merge any of these categories. Of the respondents who selected the response “unable to answer,” about 90 % of the responses were due to poor health.

#### Successful aging indicators

Following a common practice in the literature of successful aging as noted earlier, we used following outcomes as proxies of successful aging: physical function, cognitive function, self-rated health, and self-rated life satisfaction, all of which have been previously used as proxies of successful aging in the literature [42, 49].

Activities of daily living (ADL) and instrumental activities of daily living (IADL) were used to measure physical function. ADL was measured by self-reported conditions on whether the respondent needed assistance in performing six daily activities, namely bathing, dressing, indoor transferring, toileting, eating, and continence. A respondent was considered ADL independent if he or she did not need any assistance in performing any of six tasks at the time of a survey, and ADL dependent otherwise. IADL was measured by self-reported conditions at the following activities: (a) visiting neighbors, (b) shopping, (c) cooking, (d) washing clothes, (e) walking one kilometer, (f) lifting 5 kg, (g) crouching and standing up three times, and (h) taking public transportation. Choices for each item were: “able to do without help,” “need some help,” and “need full help.” In a similar vein, we dichotomized IADL into independent (did not need any help in performing any of eight IADL items) and dependent (otherwise). The ADL scale was adopted from the Katz scale [69], while the IADL scale was adopted from the Lawton scale [70].

Cognitive function was measured by the Mini-mental Status Examination (MMSE), which included six domains of cognition (orientation, reaction, calculation, short memory, naming, and language) with a total score of 30. A respondent was considered as cognitively unimpaired if his/her MMSE score was 24 or above [71]. Given the low level of educational attainment of most older Chinese people, an alternative criterion of a score of 18 was applied to respondents with no education to test the sensitivity of different cut-off points for defining cognitive impairment, and very similar patterns and conclusions were obtained (available upon request). The Chinese version of MMSE used in the CLHLS is culturally translated from the internationally standard version of the MMSE questionnaire [71]. Its validity and reliability were carefully tested in pilot surveys and were verified in each wave of the CLHLS [67].

Self-rated overall health was based on a single item: “what is your overall health at present?” and self-rated overall life satisfaction was also based on a single item: “what is your life satisfaction at present?” Both were dichotomized into as good/very good (coded as 1) versus others (coded as 0). These two indicators mainly reflect subjective health conditions, whereas the ADL IADL, and MMSE indicators mainly reflect objective health conditions.

#### Covariates

To obtain robust results, we followed a common practice [14, 42, 72, 73] to control for several sets of covariates that have been shown to have an impact on self-perceived uselessness and successful aging. These covariates included demographics, socioeconomic status, family/social support, and health practices. Demographic covariates included age, sex (males vs. females), residence (urban vs. rural), and ethnicity (Han vs. non-Han). Socioeconomic status included years of schooling (0, 1–6, and 7+), lifetime primary occupation (professional/administration vs. others), and economic independence (having a retirement wage/pension and/or own earnings vs. no). Family/social support included marital status (currently married vs. not) and co-residence with adult children (yes vs. no). Health practices were measured by currently smoking (yes vs. no), currently consuming alcohol (yes vs. no), and regularly exercising (yes vs. no). As optimism has been evidenced to be associated with self-perceived uselessness and health outcomes [73, 74], a dichotomous variable of optimism “Do you look at the bright side of things?” (always/often vs. others) was further adjusted to account for some possible effects of negative or positive psychological tendencies. We also controlled the year of the survey to account for time variation.

#### Analytical strategy

Binary logistic regression models were employed to examine associations between self-perceived uselessness and successful aging. To use all possible information collected in each wave from 2000 to 2011/2012, all variables (including outcomes, self-perceived uselessness, and covariates) were considered as time-varying variables and updated with the data from each wave. Furthermore, to increase the robustness of results, we pooled these five waves together and focused only on those who were ADL independent, IADL independent, cognitively unimpaired, in good self-rated health, and having good life satisfaction at the time of the survey. Specifically, we examined whether self-perceived uselessness among whose who were ADL independent at the 2000, 2002, 2005, or 2008/2009 wave was associated with a reduced likelihood of subsequent ADL independence two or three years later in the 2002, 2005, 2008/2009, or 2011/2012 waves. We performed the same analyses for the other four successful aging indicators. In all models, covariates were included. Given the multiple observations for the same individual, we adjusted the intra-person correlation in each model.

Because of such analytical strategies, the valid sample size of observations in the present study varied depending on different health outcomes from 7,180 individuals with 10,523 observations in the case of the IADL independent model, to 16,241 individuals with 25,212 observations in the case of the ADL independent model. A subset of identical analyses was performed for the oldest-old population aged 80 or older. The sample size of the oldest-old in the analyses ranged from 2,672 individuals with 3,095 observations in the case of the IADL independent model to 11,094 individuals with 15,196 observations in the case of the ADL independent model.

The proportion of missing values for all variables in the analysis was less than 2 %. To reduce possible biases due to missing values in the analyses and inferences, we employed multiple imputation techniques for all variables. Other alternative imputations such as modes were also used to test the sensitivity of imputations and produced very similar results. We did not apply sampling weights in the regression models because the CLHLS weight variable was unable to reflect the national population distributions with respect to variables other than age, sex, and urban or rural residence [75]. The results from unweighted regression models produce unbiased coefficients when including variables related to sample selection (i.e., age, sex, and urbanicity) [76]. Weighted regressions would unnecessarily enlarge standard errors [77]. All analyses were performed using Stata version 13.1.

## Results

Table 1 lists the sample distribution and the observed proportion of always having self-perceived uselessness by study variable. The total sample in Table 1 only included those in Fig. 1 that had at least one follow-up, consisting of 19,070 individuals with 29,954 observations. The percentage distributions in Table 1 were estimated from observations.

**Table 1:** Sample distribution by study sample, CLHLS 2000, 2002, 2005, and 2008/2009

Variables	% ^a^	Self-perceived uselessness categories (percentages)					
		Always	Often	Sometimes	Seldom	Never	Unable to answer
Total (*n*)	29,954 ^b^	6.8	14.2	34.1	19.9	18.6	6.4
Successful aging indicators at follow-ups							
ADL dependent	26.9	8.0	16.2	32.6	16.0	13.9	13.3
ADL independent	73.1	6.3	13.4	34.6	21.4	20.4	4.9
IADL dependent	69.1	7.5	15.6	33.9	18.5	15.8	8.7
IADL independent	30.9	5.1	10.9	34.3	23.1	25.3	1.3
Cognitively impaired	39.8	7.8	15.9	33.7	16.6	13.5	12.5
Cognitively unimpaired	60.2	6.1	13.0	34.3	22.1	22.1	2.4
Not good life satisfaction	45.1	7.7	15.4	34.7	18.4	15.6	8.2
Good life satisfaction	54.9	6.0	13.1	33.6	21.2	21.1	5.0
Not good self-rated health	57.5	7.4	15.5	35.1	18.4	15.6	8.0
Good self-rated health	42.5	5.9	12.3	32.6	22.0	22.9	4.3
Covariates							
Age (years) (mean)	83.6 ^c^	--	--	--	--	--	--
Women	55.2	7.5	16.0	35.0	18.2	15.2	8.1
Men	44.8	5.8	11.9	33.0	22.0	22.9	4.4
Rural	54.5	7.6	15.7	34.8	19.6	15.2	7.1
Urban	45.5	5.7	12.3	33.2	20.3	22.9	5.6
Non-Han ethnicity	6.6	3.6	10.9	37.0	22.9	19.0	6.6
Han ethnicity	93.4	7.0	14.4	33.9	19.7	18.6	6.4
Non-white collar occupation	91.1	6.9	14.8	34.7	19.7	17.1	7.8
White collar occupation	8.9	4.8	7.9	28.1	21.9	34.4	2.9
Economic dependence	67.2	7.3	15.8	35.1	18.9	14.5	8.4
Economic independence	32.8	5.5	10.9	32.1	21.9	27.1	2.5
0 years of schooling	58.4	7.7	16.4	34.8	18.3	14.3	8.6
1–6 years of schooling	30.8	5.8	12.0	34.4	22.2	21.8	3.9
7+ years of schooling	10.8	4.3	8.3	29.8	22.1	33.4	2.2
Currently not married	62.5	7.3	15.5	34.3	18.4	15.7	8.7
Currently married	38.5	5.8	12.0	33.7	22.3	23.4	2.8
Non-coresidence with children	38.9	7.6	15.1	34.3	19.4	20.0	3.5
Coresidence with children	61.1	6.2	13.5	33.9	20.2	17.8	8.3
Currently nonsmoking	79.4	6.9	14.5	34.1	19.4	18.1	7.0
Currently smoking	20.6	6.1	12.9	34.0	21.6	21.1	4.4
No current alcohol consumption	78.4	7.1	14.8	34.2	19.4	17.6	6.9
Current alcohol consumption	21.6	5.5	11.8	33.6	21.8	22.6	4.8
No regular exercise	64.2	7.7	16.2	35.3	18.3	14.5	8.0
Regular exercise	35.8	5.1	10.5	32.0	22.7	26.2	3.6
Not optimistic	25.6	8.8	18.5	32.3	12.3	7.3	20.8
Optimistic	74.4	6.1	12.7	34.7	22.5	22.6	1.5
Wave 2000	19.4	5.2	12.4	34.3	20.3	16.1	11.7
Wave 2002	27.9	8.3	14.6	33.4	17.4	15.6	10.7
Wave 2005	25.1	8.4	15.0	32.3	16.8	16.0	11.5
Wave 2008	27.6	7.2	16.6	29.0	17.1	14.9	15.2

^a^refers to percentages unless otherwise stated. All percentages in the table refer to observations of 29,954 from 19,070 individuals who had at least one follow-up from 2000 to 2011/2012. ^b^sample size; c, mean age. ^c^ --, not applicable. The distributions of 19,070 individuals at their baseline are similar to what are presented in the table. All distributions refer to the pooled data from 2000 to 2008/2009

The proportions for always, often, sometimes, seldom, and never experiencing self-perceived uselessness were 6.8, 14.2, 34.1, 19.9, and 18.6 %, respectively. About 6.4 % were not able to answer the question. Among the 29,954 observations, about 73 % were ADL independent, 31 % were IADL independent, 60 % were cognitively unimpaired, 55 % had good life satisfaction, and 43 % had good self-rated health at baseline.

The upper panel of Table 2 presents odds ratios and their 95 % confidence intervals of successful aging for frequencies of self-perceived uselessness in the presence of a rich set of covariates. The results clearly showed that self-perceived uselessness was negatively associated with these five successful aging indicators. Among those who were ADL independent at baseline, compared to those who never had a self-perceived uselessness, those who always had such a perception of uselessness were 43 % (=1–0.57) (*p* < 0.001) less likely to remain in the ADL independent state two or three years later. The corresponding figures for those who often or sometimes had such a feeling were 35 % (*p* < 0.001) and 23 % (*p* < 0.001), respectively. There was no difference in subsequent ADL independence between older adults seldom having a feeling of uselessness and those never having such a feeling.

**Table 2:** Associations between self-perceived uselessness and subsequent successful aging indicators, CLHLS 2000–2011/2012

	Successful aging indicators				
	Remaining ADL independent in a subsequent wave for those who were ADL Independent at a wave	Remaining IADL independent in a subsequent wave for those who were IADL Independent at a wave	Remaining cognitively unimpaired in a subsequent wave for those who were cognitively unimpaired at a wave	Remaining good life satisfaction in a subsequent wave for those who were reported good life satisfaction at a wave	Remaining good self-rated health in a subsequent wave for those who were good self-rated health at a wave
Ages 65+					
Self-perceived uselessness					
Always (never)	0.57*** (0.48–0.67)	0.69*** (0.55–0.86)	0.76*** (0.65–0.90)	0.76*** (0.65–0.90)	0.84* (0.71–1.00)
Often (never)	0.65*** (0.57–0.75)	0.70*** (0.59–0.83)	0.73*** (0.64–0.83)	0.83** (0.74–0.94)	0.70*** (0.61–0.80)
Sometimes (never)	0.77*** (0.68–0.86)	0.87* (0.77–0.98)	0.82*** (0.74–0.90)	0.82*** (0.75–0.90)	0.75*** (0.68–0.82)
Seldom (never)	0.93 (0.82–1.06)	0.96 (0.84–1.09)	0.95 (0.85–1.06)	0.90* (0.82–1.00)	0.88* (0.80–0.98)
Unable to answer (never)	0.50*** (0.42–0.61)	0.43*** (0.28–0.66)	0.77 (0.54–1.10)	0.61*** (0.48–0.77)	0.54*** (0.41–0.72)
N	25,212	10,523	21,140	17,568	15,462
No. of individuals	16,241	7,180	13,959	12,963	11,648
Wald chi square	1090.7***	922.0***	1531.8***	244.0***	290.6***
rho	0.20***	0.09***	0.07***	0.10***	0.10***
Ages 80+					
Self–perceived uselessness					
Always (never)	0.61*** (0.50–0.74)	0.94 (0.62–1.42)	0.76*** (0.63–0.92)	0.75** (0.61–0.90)	0.83* (0.68–1.02)
Often (never)	0.70*** (0.60–0.81)	0.84 (0.62–1.14)	0.66*** (0.57–0.76)	0.82** (0.70–0.95)	0.71*** (0.61–0.83)
Sometimes (never)	0.83** (0.73–0.94)	1.04 (0.84–1.29)	0.80*** (0.71–0.90)	0.82** (0.73–0.93)	0.75*** (0.67–0.84)
Seldom (never)	0.96 (0.83–1.11)	1.20 (0.95–1.52)	0.92 (0.81–1.04)	0.91 (0.80–1.03)	0.86* (0.76–0.97)
Unable to answer (never)	0.51*** (0.41–0.62)	0.39** (0.19–0.79)	0.62* (0.42–0.90)	0.60*** (0.46–0.78)	0.50*** (0.37–0.68)
N	15,196	3,095	11,748	11,672	10,072
No. of individuals	11,084	2,672	8,843	9,135	8,054
Wald chi square	583.0***	135.8***	713.1***	198.8***	198.9***
rho	0.16***	0.06***	0.04***	0.11***	0.09***

(1) unweighted. (2) Figures for self-perceived uselessness in the Table are odds ratios from longitudinal binary logistic regression models controlling for all variables listed in the table. (3) rho is the intrapersonal correlation of individuals across waves. (4) The “never” category is the reference category. (5)*p < 0.05, **p < 0.01, ***p < 0.001

After adjusting for possible covariates, the reduced odds ratios of remaining IADL independent were 31 % (*p* < 0.001) for those who always or often had a feeling of uselessness compared to those never having such a feeling provided that they were both IADL independent at baseline. The odds ratio would be reduced to 13 % (*p* < 0.05) for a respondent who only sometimes had such a feeling; and there was no difference between the category of seldom and the category of never.

Among the cognitively unimpaired population, older adults who always or often had a feeling of uselessness had odds of remaining cognitively unimpaired reduced by 24 % (*p* < 0.001) and 27 % (*p* < 0.001), respectively, compared to those who never had feelings of uselessness. Respondents who sometimes had a feeling of uselessness had 18 % (*p* < 0.001) lower odds of being cognitively unimpaired compared to those who never had feelings of uselessness.

In the case of the two subjective measures of successful aging, a high frequency of having a feeling of uselessness significantly reduced the odds of maintaining good life satisfaction and good self-rated health. For example, always, often, sometimes, and seldom having feelings of uselessness were associated with lower odds of maintaining good life satisfaction by 24 % (*p* < 0.001), 17 % (*p* < 0.01), 18 % (*p* < 0.001), and 10 % (*p* < 0.05), respectively, compared to never having such a perception. In the case of self-rated health, these corresponding figures were 16 % (*p* < 0.05), 30 % (*p* < 0.001), 25 % (*p* < 0.001), and 12 % (*p* < 0.05).

With one exception for cognitive function, not being able to answer the question was significantly associated with reduced odds of remaining ADL independent, IADL independent, and having good life satisfaction, and good self-rated health by 50 % (*p* < 0.001), 57 % (*p* < 0.001), 39 % (*p* < 0.001), and 46 % (*p* < 0.001), respectively, compared to never having a feeling of uselessness. These results are expected because 90 % of those who were unable to answer the question were in poor health.

The lower panel of Table 2 shows that compared to the associations among the entire sample, the significance of negative associations among the oldest-old aged 80 or older was almost unchanged with one exception for the case of IADL independent model where the association was not significant.

To examine the possible moderating role of optimism in the association between self-perceived uselessness and successful aging, we further performed supplementary analyses that included interaction terms between optimism and self-perceived uselessness (not shown, but available upon request). No significant interactions were found.

## Discussion

Using a unique large and nationally representative survey of older adults in China, this study examined the association between self-perceived uselessness and successful aging. To our knowledge, the present study is among the first to investigate whether self-perceived uselessness is negatively associated with different successful aging indicators measured both objectively-oriented and subjectively-oriented indicators from a longitudinal perspective under a biopsychosocial model of successful aging in non-Western countries. In this study, we have shown that self-perceived uselessness is associated with successful aging. Specifically, self-perceived uselessness could reduce the odds of remaining in ADL and IADL independent and cognitively unimpaired statuses, and could reduce the odds of having good life satisfaction and good self-rated health. These findings are in line with most previous studies in Western societies [1–10]. Our study adds additional evidence from a non-Western society to support the argument that self-perceived uselessness is negatively associated with subsequent successful aging and that feelings of usefulness could influence trajectories of successful aging at older ages.

In a traditional Confucian society like China, seniors or older parents often hold positions of respect within the family as well as in the society; and people with long life are revered and considered a symbolic image of successful aging. Consequently, most Chinese older adults consider family life and intergenerational relations as the most important things in their daily life [78]. These viewpoints and environments could help the Chinese older adults well maintain the positive perception about their usefulness to others. As a result, prevalence of self-perception of uselessness in China could be expected to be low. However, urbanization, industrialization, individualization, demographic transition (population aging), and globalization all present challenges for older people’s traditional roles in China. Some studies show that Chinese older adults have started to perceive themselves as a burden to family and society [79]. Younger generations have also started to change their attitudes toward aging [80]. Furthermore, it is possible that the sense of purpose in life and personal growth may diminish with age among older adults [2]. All these changes may cause older adults to internalize some negative views into their self-image [81], and thus develop their self-perception of uselessness. Provided that most older adults who feel more useful are more likely to be motivated to take better care of themselves in order to ensure that they can be included with social roles and activities, and provided that the desire to play a valued and useful role in the family and society will not decrease much among most older adults [1, 2, 79], our findings imply that those who have a feeling of uselessness are likely to be vulnerable in China. More studies are clearly warranted to shed the light on what factors are associated with self-perceived uselessness to identify those most vulnerable older adults for possible interventions.

The following underlying relations about the psychological, physiological, and behavioral pathways of the influence of self-perception on successful aging may possibly explain the negative associations between self-perceived uselessness and several successful aging indicators [31, 73, 82]. Psychologically, it has been argued that individual expectations regarding ageing turn out to be self-fulfilling, resulting from beliefs about self-control beliefs and self-efficacy [3]. Self-perceived uselessness diminishes beliefs about self-control and self-efficacy that lead to a low resilience capacity and depression, preventing from psychological well-being [1, 2]. On the other hand, greater perceived usefulness could lead to a positive appraisal of capacity to deal with daily adversity or difficulties [2]. Physiologically, neuroendocrine and neurohumoral changes, immune alterations, autonomic and cardiovascular dysregulation, or central neurotransmitter system dysfunction could be attributable to cardiovascular diseases and subsequently other new symptoms and disabilities in older age [82, 83]. Behaviorally, as views on or attitudes toward aging have the potential to activate responses to external triggers (e.g., incidence of a disease or other experiences in physical deterioration) [28], self-perceived uselessness likely leads to less optimal healthcare seeking behaviors [84] and less engagement in preventive and health-promoting activities [85], thus subsequently influencing one’s health or leading to more rapid declines in health [73]. Some researchers have even argued that the associations between self-perceived uselessness and health outcomes may be due to the way in which a person reacts to poor or deteriorating health [73].

Another important finding of the present study is that the negative associations between self-perceived uselessness and successful aging still hold at the oldest-old with an exception for IADL disability. Some studies in Western societies showed increased risks of disability for older adults with feeling of uselessness from their 60s and 70s into 70s and 80s [1]. We extended such findings by showing that always or often having a feeling of uselessness is associated with lower odds of ADL and IADL functioning within the oldest-old aged 80 years or older. The oldest-old may be a selectively more robust group in physical and cognitive functions as well as in psychological resilience compared to their same cohort peers who did not survive to oldest-old ages. Indeed, many of the oldest-old are psychologically as resilient as younger aged cohorts, although their physical and cognitive functions were at a disadvantage compared to the younger cohorts [86]. This may be due to many of the oldest-old having realistic outlooks, making needed adaptations to the changing environments, and having developed adequate coping strategies [53]. In this regard, for achieving successful aging, the findings among the oldest-old have more theoretical and practical significance than the findings among the general older population. However, they are inadequately studied in the existing literature. We call for more research to focus on this growing subpopulation.

One strength of the present study is the large size of the study sample, which included nearly 7,000 older adults with 10,523 observations in the case of IADL models, and included 16,241 older adults with 25,212 observations in the case of ADL models. So far, no studies have ever contained such a large sample size to investigate the association between self-perceived uselessness and successful aging. Another strength is the longitudinal nationally representative multi-wave examination from a transitional and non-Western society. The longitudinal analyses largely eliminated the potential bidirectional relationships between self-perceived uselessness and successful aging [72], making our results more robust. A third strength is its inclusion of examinations of the association between self-perceived uselessness and successful aging among the oldest-old that are currently understudied in the literature.

Our findings have important implications. Given the negative association between self-perceived uselessness and successful aging, interventions that promote positive views of ageing, and aim to change societal views of aging seem crucial [31, 51, 82, 87]. Prior longitudinal studies have shown that the view of self-perceived uselessness among older adults could incorporate generalized views on older people by the public [88, 89]. Thus, interventions should include all stakeholders, such as governments, policy-makers, media, the public, medical and social service professionals, families, and individuals to disseminate knowledge about old age to maximize the influence of positive views on aging in older individuals’ everyday lives [31]. Older people have made enormous contributions to society, and continue to do so. Awareness of and intentionality of aging processes must be understood among older adults themselves, and increased dialogues between generations and different groups of populations would also likely be effective for current and future generations of older people.

This study has the following limitations. First, self-perceived uselessness was measured by a single item. Multi-item measures of uselessness would likely more completely reflect the perception of uselessness [10]. We encourage additional studies to investigate more sophisticated, positive/negative, and/or domain-specific constructs of self-perceived uselessness—and self-perceived aging more generally— to better understand its linkage to mortality and the mechanisms of successful aging [1, 87]. Second, we did not examine whether there is an association between changes in self-perceived uselessness and subsequent successful aging. Although previous studies showed that self-perceived uselessness is relatively stable [2, 10], changes are still frequent [1]. It would be interesting to investigate and identify those who had changes and who did not. Third, some important covariates such as depression were not available in the CLHLS, which may introduce some biases. Fourth, we did not include the respondents’ perceptions about successful aging due to the availability of data. Although we have included life satisfaction and self-rated health as two subjectively measured indicators of successful aging, the concept of successful aging encompasses much more than just these two indicators [34, 45]. A growing body of researchers are currently including laypersons’ perceptions about successful aging as core part of the biopsychosocial model of successful aging. Therefore, it is likely that the association between self-perceived uselessness and successful aging is incompletely understood at this time. More studies including more comprehensive measures of both self-perceived uselessness and successful aging are clearly warranted. Fifth, it would be interesting to know what kinds of characteristics are associated with such feelings of self-perceived uselessness, which could help identify the most vulnerable subpopulation in need of intervention. Finally, individuals in different cultures may develop different views on self-perceived uselessness, which were not compared in the current study. Much work remains to fully utilize the opportunity to intervene and improve the lives and health outcomes of older people as they age.

## Conclusions

Based on a unique large nationwide representative survey of older adults in contemporary China, this study found that self-perceived uselessness is negatively associated with the odds of maintaining in ADL and IADL independent status, cognitively unimpaired status, good life satisfaction, and good self-rated health. These negative associations are valid among both the entire older population and the oldest-old. We conclude that feelings of usefulness are crucial in achieving successful ageing. Our findings could be informative for China in the development of public health programs that aim to overcome negative self-perceptions about aging and promote successful aging.

## Abbreviations

ADL: Activities of daily living
CLHLS: Chinese Longitudinal Healthy Longevity Survey
IADL: Instrumental activities of daily living
MMSE: Mini-mental status examination
